# Supplementary material for: A Mixed-Methods Participatory Intervention Design Process to Develop Intervention Options in Immediate Food and Built Environments to Support Healthy Eating and Active Living among Children and Adolescents in Cameroon and South Africa
Source: Int J Environ Res Public Health. 2022 Aug 18;19(16):10263. doi: 10.3390/ijerph191610263 (PMC9408217; doi:10.3390/ijerph191610263)
Supplement: Supplementary file 1 [file ijerph-19-10263-s001.zip › ijerph-1814624-supplementary.pdf]

Table S1. Data extraction matrix template.

				Capability		Opportunity		Motivation		
**Driver**	**Level**	**Explanation**	**Barrier/Facilitator**	**Psychological**	**Physical**	**Physical**	**Social**	**Automatic**	**Reflective**	**Illustrative Quote**
E.g., Sale of unhealthy food	School environment	Availability of unhealthy food at the tuck-shop enables unhealthy eating	Barrier			x				“We also had difficulty in the kind of things that are sold, for example, lots of chips are sold and lots of pies are sold and the white bread you see, so there are a lot of carbs that are wrong” (Primary school Principal, Johannesburg)
